# Quality of information of websites dedicated to obesity: a systematic search to promote high level of information for Internet users and professionals

**DOI:** 10.1007/s40519-020-01089-x

**Published:** 2021-03-04

**Authors:** Alessandra Perra, Antonio Preti, Valerio De Lorenzo, Antonio Egidio Nardi, Mauro G. Carta

**Affiliations:** 1 Department of Health Sciences and Public Health, University of Cagliari, Cagliari, Italy; 2 PRoMIND, Services for Mental Health SRLS, Rome, Italy; 3 Federal University of Rio de Janeiro, Rio de Janeiro, Brazil

**Keywords:** Obesity, Weight-loss, Health literacy, Information dissemination, Internet, Quality

## Abstract

**Background:**

The Internet is increasingly used as a source of information. This study investigates with a multidimensional methodology the quality of information of websites dedicated to obesity treatment and weight-loss interventions. We compared websites in English, a language that it is used for the international scientific divulgation, and in Italian, a popular local language.

**Methods:**

Level of Evidence: Level I, systematic review search on four largely used search engines. Duplicated and unrelated websites were excluded. We checked: popularity with PageRank; technological quality with Nibbler; readability with the Flesch Reading Ease test or the Gulpease readability index; quality of information with the DISCERN scale, the JAMA benchmark criteria, and the adherence to the Health on the Net Code.

**Results:**

63 Italian websites and 41 English websites were evaluated. English websites invested more in the technological quality especially for the marketing, experience of the user, and mobile accessibility. Both the Italian and English websites were of poor quality and readability.

**Conclusions:**

These results can inform guidelines for the improvement of health information and help Internet users to achieve a higher level of information. Users must find benefits of treatment, support to the shared decision-making, the sources used, the medical editor's supervision, and the risk of postponing the treatment.

**Supplementary Information:**

The online version contains supplementary material available at (10.1007/s40519-020-01089-x).

## Introduction

The prevalence of obesity in the last 50 years has increased and it is considered today as a serious major health challenge worldwide [5]. When measured in terms of body mass index (BMI = a person’s weight in kilograms divided by their height in meters squared), the prevalence of people with obesity (BMI ≥ 30 kg/m^2^) ranges from 3.7% in Japan to 38.2% in the United States [5]. Moreover, the percentage of people with obesity is increasing, especially in North Africa and the Americas. In United States, the 42.4% of the adult populations has an obesity condition [22]. In Italy, the 54.1% of the adult population has an overweight condition and just 19.8% has an obesity condition. Indeed, obesity might represent a milder problem in Italy than in other European countries, but the childhood prevalence is high: 36% for boys and 34% for girls [32, 41]. Obesity is a chronic condition that depends on many factors, principally physical inactivity and overeating; additional risk factors for obesity include medications, psychological factors, disease and social issues [31]. In turn, obesity represents a risk factor for severe metabolic, cardiovascular, degenerative and neoplastic diseases, influencing a sensible decline in life expectancy. It also impacts on the quality of life, increasing the risk of unemployment, hampering socio-economic productivity, and causing a greater occurrence of social disadvantage and disability. To manage such complex and multifactorial condition, it is necessary applying prevention measures such as the promotion of healthy behaviors and health-related treatments [3, 40].

Currently, several international medical organizations have developed specific guidelines for the treatment of obesity [35]. Clinical guidelines are structured recommendations developed to orient health professionals on appropriate health care and health information promotion [26]. They include all the information about the medical condition, the consequences of the condition on health and daily living, the different treatments, also specifying benefits and risk (indications and contraindications, special attention is dedicated to involving patients in the decision-making process [34]. All these components should be present in the correct health information in order to promote high-quality information to the people that are experiencing the condition. Health professionals, administrators, and funders should use guidelines as tools that can reduce the gap between clinical routine and scientific evidence [20], and guarantee the right of equal access and the right of high standards of treatment.

The various guidelines on obesity emphasize the importance of a multidisciplinary approach and the needing to intervene earlier, in primary care settings, with lifestyle intervention [24]. The lifestyle intervention includes the diets for weight loss and physical activity. However, those interventions will only be effective under intensive behavioral therapy approaches. The guidelines underline the chronic nature of obesity and the need for long-term care and the importance of a comprehensive approach to a lifestyle change [35]. For this reason, it is discouraged the proposal of a “magic diet”, which often can have adverse effects. For the physical activity depending on a severity level, it is necessary to consult a health professional. Physical activity alone does not have efficacy in promoting long-term weight loss but favor the loss of fat mass and reduce the health risk related to obesity [15]. Most guidelines support 200–300 min per week for long-term weight loss and moderate-intensity physical activity between 150 and 250 min per week to be effective to prevent weight gain, although that intensity will provide only modest weight loss. All guidelines consider pharmacotherapy as an adjunct treatment to lifestyle changes, especially when BMI > 27 in association with 1 or more obesity comorbidities or when BMI > 30, with or without metabolic consequences [19]. Pharmacotherapy for obesity supports diets and in combination with behavioral therapy leads to improve significantly weight loss than either alone. There is a consensus that the patient should be informed about the available drugs and their risk (side effect) and benefits [2]. For people with obesity with BMI greater than or equal to 40 or BMI greater than or equal to 35 with obesity-related comorbid conditions, an alternative is bariatric surgery. Bariatric surgery leads to substantial long-term weight loss, improves many obesity-related comorbid conditions, and reduces mortality [24]. It is considered important that the patients are carefully informed about the complications of the surgery.

In the Digital Era, the access to information about the treatment and the healthy behaviors is rapidly changing, and the individuals searching health information on the Internet have an active role in their health management [13]. It has been estimated that there were 4.39 billion Internet users worldwide in 2019 [21, 25], of whom 312.3 million (95% of the total American population) were from American countries, and 461.2 (90% of the total European population) were from European Union. Some studies that were carried out in the U.S. have found that up to 55% of the Internet population have used the Web to get health or medical information, and the 70% of those who search health information admit that the Web information influences their decision about how to treat an illness or condition [18]. Currently, the evidence points to the Internet as a hugely used tool for the sharing of medical knowledge and its use is increasing [13]. The practice of shared decision-making between professionals and consumers has the objective to find the best treatment choice for a person [9]. This aspect can have a profound effect on health-related decisions. Indeed, health information on Internet may make patients better informed about health outcomes, services resources, and access thanks to the easy updating process and the potential for interactive formats that promote easy understanding of information [30]. However, health information on the Internet may be inappropriate, or of poor informative quality, misleading, or misinterpreted [17]. The quality of health information available online for healthcare users should not be ignored, because of the important role that has in the health promotion [4, 16]. However online health information on websites, in general, are inconsistent in quality, especially for reliability. Moreover, the information is often incomplete and the authors or references are omitted [17, 29]. The quality of information about obesity treatment and management on the Internet is not known. Because of the health and economic impact of obesity, it is important to understand better the quality of the online information on this condition. Evaluating the quality of Internet information can be an important way to offer support for Internet users about which kind of information they have necessary to find to promote high-quality level and equity access to online information.

### Aims

This study set out to investigate the readability, accessibility and technological usability, and the quality of information of websites devoted to spreading information about the treatment of obesity and interventions on weight loss in people with obesity. In particular, due to the large usage of the Internet worldwide, we were aimed to comparing websites in two different languages, one language that is used both as a local language and also as a language for the international scientific divulgation (English) and one that represents mainly a popular local language (Italian).

## Methods

A systematic search was conducted used the three most common commercial search engines “Google” (www.google.com), “Bing” (www.bing.com), “Yahoo” (www.yahoo.com), and one independent search engine “Duck Go” (duckduckgo.com/), which aims at preserving the privacy of the users. These search engines share more than the 98% of all searches worldwide and across the platforms, such as desktop, tablet, or mobile [37].

The following key terms were used: “Obesity therapy/Obesity weight loss” in English and “Terapia obesità/Dimagrimento obesità” in the Italian language. The websites were selected in order of appearance, the first 20 for each English and Italian key term in each search engine. There is evidence that users concentrate their exploration of the websites that are retrieved from a search engine to the first ten entries and rarely go beyond the first two pages of the results [23]. Discussion or forums websites, websites requiring password or payment, not written document (video, only title text, advertisement), and scientific articles were excluded. The search engines were assessed from March 16th to 31st 2020. The evaluation was done blind with the average of the results of two researchers. Popularity of the website was checked via Google’s PageRank with https://checkpagerank.net/ (Google’s page rank), Google’s PageRank is one of the methods Google uses to determine a page's relevance or importance [11].

Technological quality of the website was checked with Nibbler at https://nibbler.silktide.com/ using the following indexes: overall; accessibility; experience; marketing; technology; mobile. Each website was assessed for its accessibility (such as ease of locating information on the website, URL format, and page titles), the rated user experience (such as the content value, format, mobile availability, internal links, etc.), the marketing (links to social media, popularity, meta tags, freshness, etc.) and the quality of informatics used [1].

Readability for the English websites was assessed using the Flesh Kincaid Reading Ease and the Flesh Kincaid Grade Level, tested with the readability test tool of WebFX at the following address: https://www.webfx.com/tools/read-able/. The Flesch Reading Ease score takes into account factors such as the number of words per sentence and the number of syllables per word to give a score from 0 to 100, with a high-scoring text being more easily understood than one with a low score. A text with a score of 71–100 is considered ‘easy’ to read, with the average 11-year-old able to read it with ease. A score of 61–70 is considered of ‘standard’ difficulty, with children aged 13–15 years being able to read it. A text with a score of 60 or below is considered ‘difficult’ to read [14, 28].

Readability for the Italian websites was assessed using the Gulpease readability Index [27], testing at the following address https://farfallaproject.org/readability_static/. The Gulpease index takes into account the length of a word in characters rather than in syllables, which proved to be more reliable for assessing the readability of Italian texts. The index ranges from 0 that means lowest readability to 100 maximum readability [39]. Since the Flesh Kincaid reading test and the Gulpease readability Index, which is tailored for the Italian language, are not directly comparable, readability has been compared between languages on the degree of complexity. The degree of complexity has been grouped into three class: “easily readable” (for texts that the average 11-year-old should be able to read); “standard level of readability” (for texts that children aged 13–15 years old should be able to read); and “difficult to read” (for texts that require high school or a even higher level of literacy).

Quality of the information provided by the website at the specific web page dedicated to the topic was assessed with the DISCERN scale, the JAMA benchmark criteria, and the adherence to the Health on the Net code (HONcode). DISCERN is an instrument designed to help users of consumer health information judge the quality of written information about treatment choices [9]. It is consisting of 16 items, each criterion is rated on a scale from 1 to 5. Higher is the level better is the quality of information. It is dived into three main sections assessing the reliability, whether it can be trusted as a source of information about treatment choice; the quality of information; and the overall quality [10, 28]. The JAMA benchmark criteria range from 0 to 4, and it is aimed at to critically judge the credibility, reasonability, and utility of medical information read on the Internet [36]. The JAMA benchmark criteria assess the following core standards: website authorship had to formally include authors, contributors, affiliations, and credentials,attribution should include references and sources used for the content, and copyright information; disclosures should include details about sponsorship, advertising, commercial funding, potential conflicts of interests; currency should include the date of posted and updated information [36]. The HONcode certification was proposed by the Health On the Net Foundation (HON) and certificates the quality of the medical information provided on the Internet [6].

### Statistics

All data were coded in Excel and analyzed using the Statistical Package for Social Sciences (SPSS) version 20. Additional analyses were carried out in R [33]. All tests were two tailed. Due to the explorative nature of the analysis, significance threshold was set at *p* < 0 0.05. Means with standard deviations were reported for continuous variables. Counts and percentages were reported for categorical variables. Continuous variables were compared between groups with a non-parametric test (Mann–Whitney *U* test). Chi-square tests or Fisher's exact tests were used to analyze categorical data. Intra-rater reliability for the JAMA benchmark criteria and the DISCERN scale was assessed with the intraclass correlation coefficient (ICC), with 95% Confidence Interval (CI). ICC values ≥ 0.60 are considered acceptable [8].

## Results

**Fig. 1:**
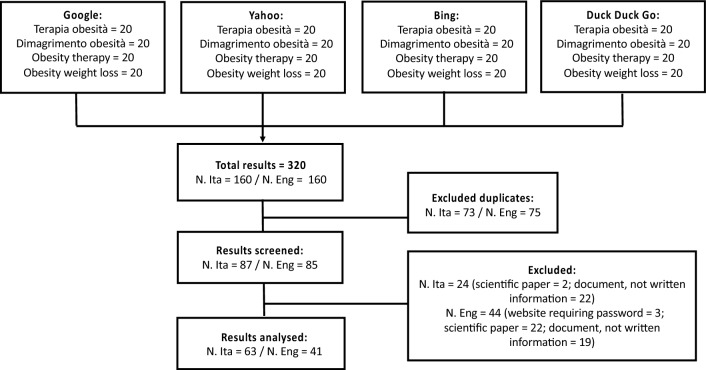
Flowchart of search results, with all steps of the procedure

The initial sample consisted of 320 websites (160 Italian websites and 160 English websites). We excluded 73 Italian duplicates websites and 75 English duplicates websites. At the end of the screening we excluded 24 Italian websites (2 scientific paper, 22 document or not written information) and 44 English websites (3 requiring password, 22 scientific paper, 19 document or not written information and 5 not pertinent). Finally, we analyzed 63 Italian websites and 41 English websites (Fig. 1). Table 1 in ESM lists the main characteristics of the analyzed websites.

The key terms retrieved more “Obesity weight loss” related English websites than “Obesity therapy” related English websites. The reverse was found for the Italian websites. The percentage of retrieval by key terms did not violate the expected 50% probability at the binomial test (*p* > 0.20 in both comparisons). Google contributed to about half of retrieved websites, but the other search engines contributed as well with a percentage of unique websites (websites that were not listed in the other search engines), with no difference by language. As far as the Google rank of the retrieved websites was concerned, those in the English language had a higher rank than those in the Italian language, hence they were more popular, likely a reflection of the greater fraction of English-speaking users among Internet users (see Table 1 in ESM for details).

### Technological quality of the websites

Overall, the technological quality of the websites concerning the treatment of obesity was good, with a global average Nibbler score above 80% (Table 1 in ESM).

English websites provided a better experience of use, did a more appropriate use of marketing and were more tailored for use with mobile than the Italian websites. No differences were found in the accessibility to the site or the use of technology.

### Readability

Readability, as measured with the Flesh Kincaid test or the Gulpease readability index, suggested that the websites were on average difficult to read. Italian websites were on average more difficult to read than the English websites (see Table 1 in ESM for details).

#### Information quality: adherence to the HONCode

Only a minority of the assessed websites bothered to acquire a certification of adherence to the HONCode. English websites were more likely to present the certification of adherence to the HONCode than the Italian websites (see Table 1 in ESM for details).

#### Information quality: JAMA benchmark criteria

**Fig. 2:**
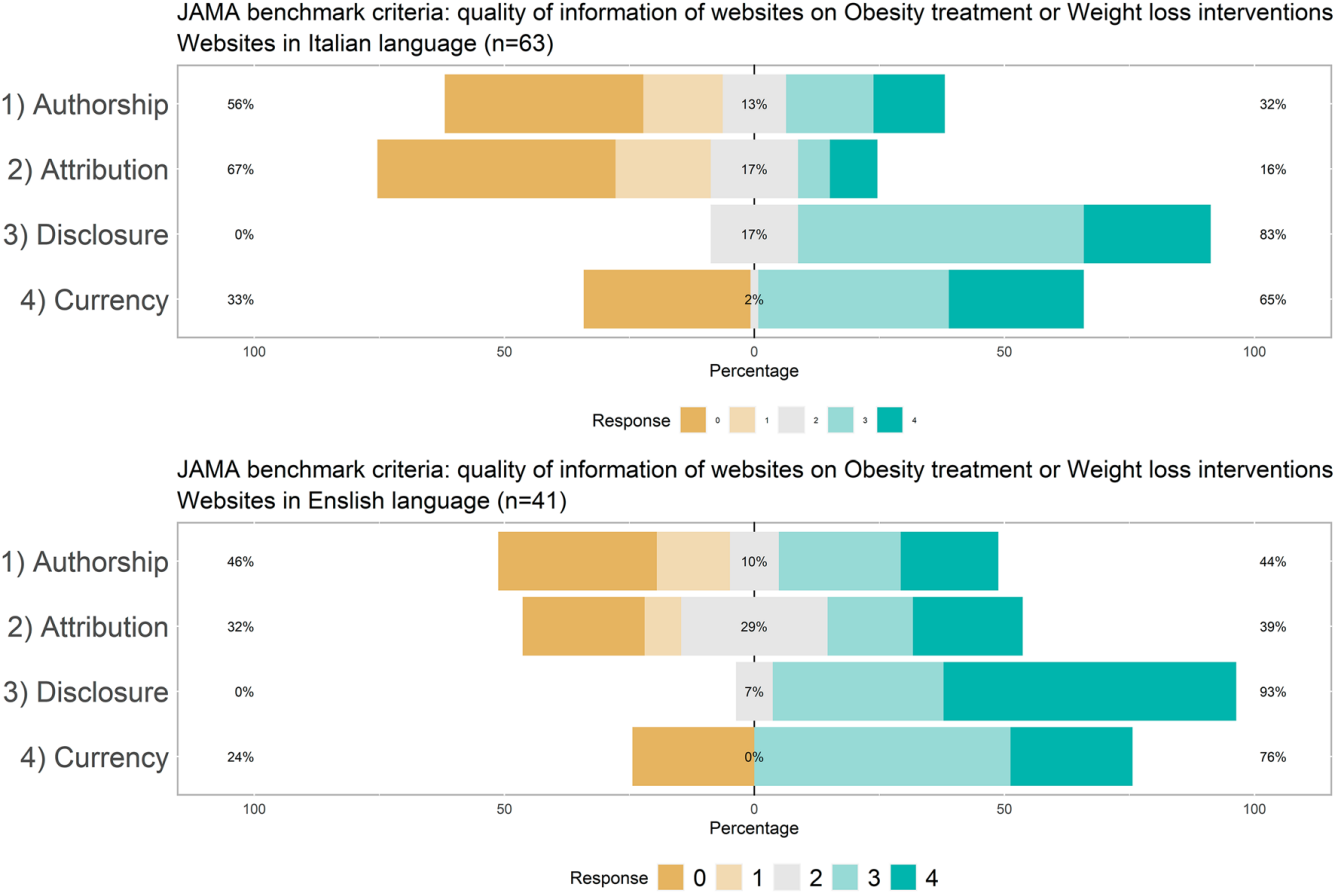
Distribution of the adherence to the JAMA benchmark criteria in Italian (top) and English (bottom) websites

Intra-rater reliability for the JAMA benchmark criteria was poor: ICC = 0.497; 05% CI = 0.319–0.638). Thus, results concerning the JAMA benchmark criteria should be considered with caution. The JAMA benchmark criteria were rarely complied with, especially for “authorship” and “attribution”, which rarely were observed in both the Italian and the English websites at a level of much (3) or completely (4) satisfied (Fig. 2). The criteria of “disclosure” and “currency” were more often met. English websites were more likely to satisfy the “attribution” and “disclosure” criteria than Italian websites (details in Table 1 in ESM).

#### Information quality: DISCERN scale

Intra-rater reliability for the DISCERN scale was excellent: ICC = 0.935; 05% CI = 0.915–0.952).

**Fig. 3:**
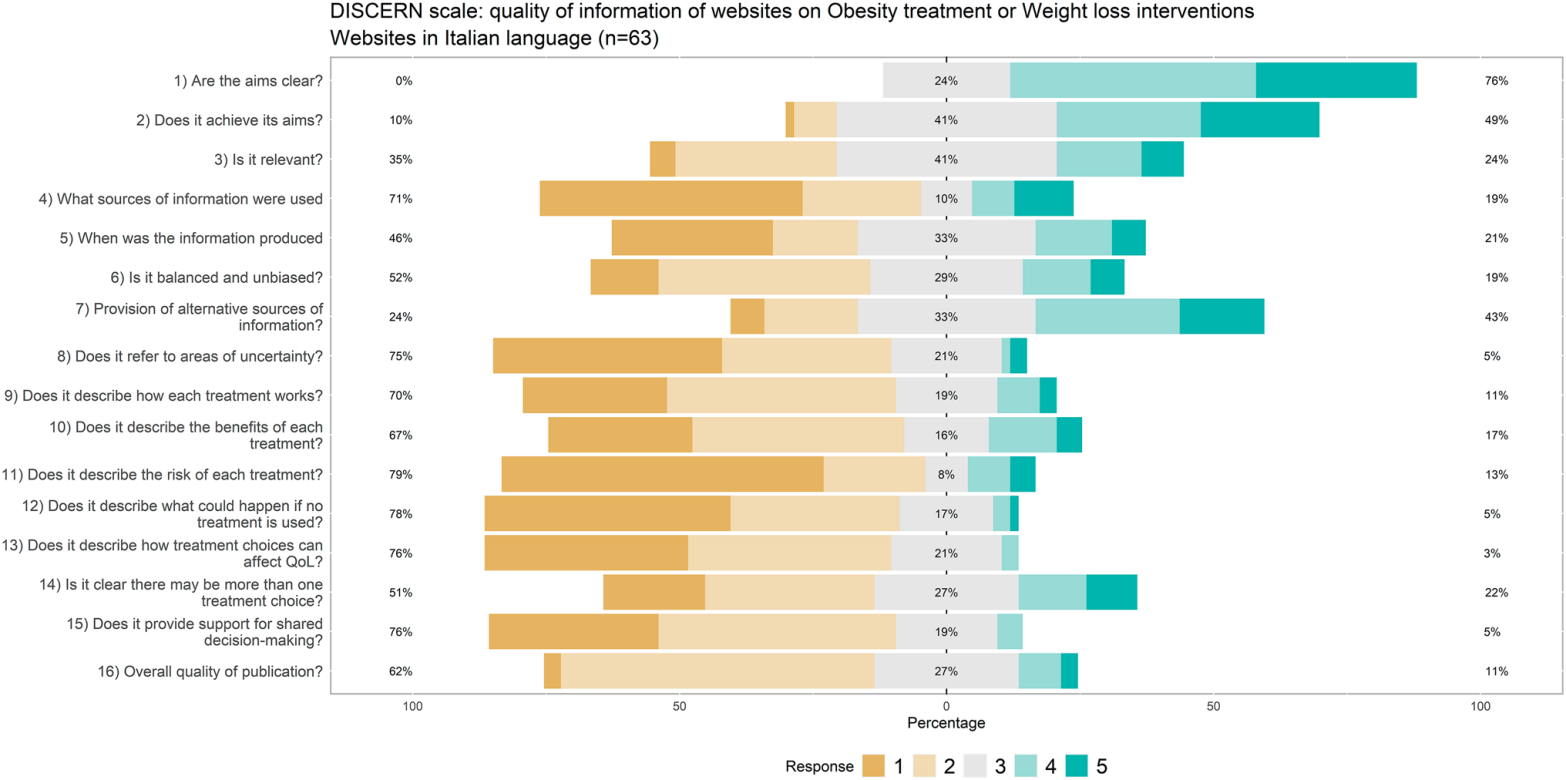
Distribution of DISCERN-scores combined and averaged across English websites (1 = low, 5 = high). Adapted from Charnock et al. [9]

**Fig. 4:**
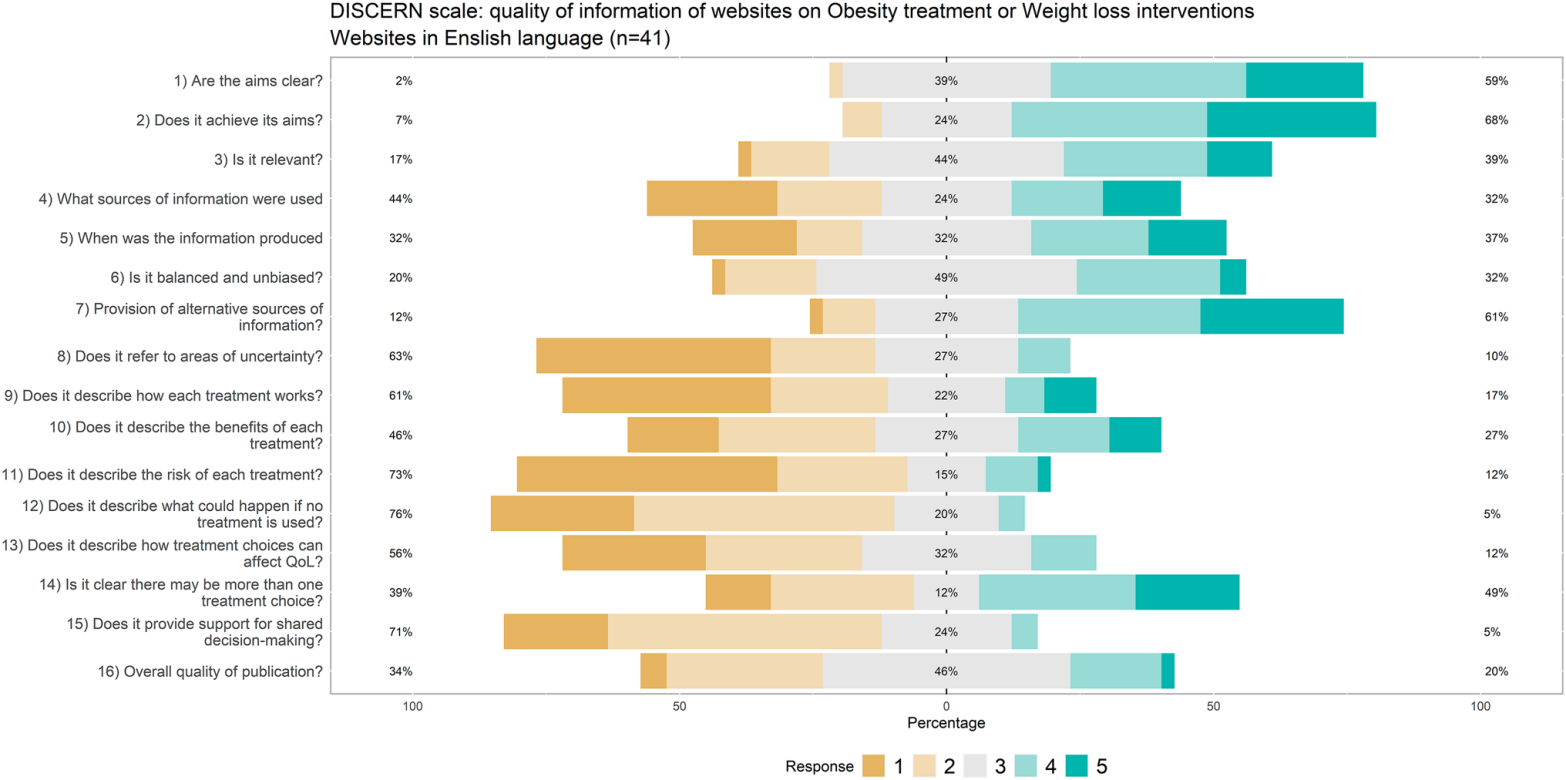
Distribution of DISCERN-scores combined and averaged across Italian websites (1 = low, 5 = high). Adapted from Charnock et al. [9]

English websites were likely to achieve a score of 4 or 5 on the DISCERN items, with some limitations about uncertainty (item 8), risk of treatment, no treatment and support for shared decision-making (items 11, 12 and 15). Up to 20% of the websites had an overall quality score of 4 or 5 (Fig. 3). Italian websites had a poorer performance on the DISCERN scale, and on items pertaining to the treatment (pro and cons, risk and so on), rarely met the best score of 4 or 5 (Fig. 4). According to the DISCERN scale, the reliability and overall quality of information were estimated to be better in English websites than in Italian websites discussing obesity treatment or weight loss interventions (see details in Table 1 in ESM).

## Discussion

This study highlights which kind of information is detailed in the Internet websites dedicated to obesity treatment for online users to achieve high-quality information promotion. To have a better grasp of this topic, we evaluated the quality information on different websites in a language used also for international divulgation and a popular language and we focused on the quality level and how the differences in quality can impact on the information goals. English websites regarding information about the treatment of obesity and interventions on weight loss in people with obesity were, in general, better than the Italian websites. In particular, the Italian websites were more difficult to read, although in both languages the websites were, on average, difficult to read, as seen in the past in other studies that evaluated the readability of English websites [1, 28]. English websites invested more in the experience of the user and mobile accessibility, an essential feature currently, since a large part of the web traffic pass now on mobile phones. Greater technological quality in these domains resulted in better marketing of the English websites compared to the Italian ones.

The analysis based on the JAMA Benchmark criteria showed that the Italian websites had a lower quality in terms of attribution and disclosure. In general, both the Italian and the English websites were of poor quality as far as the authorship and attribution were concerned; the adherence to the HONcode certification was also poor, with a global scarce adherence, but better results in the English websites. On the DISCERN tool, too, Italian websites had poorer performances, especially for the uncertainty, risk of treatment, risk if no treatment is used and support for shared decision making (items 8, 11, 12, 15). A lot of websites do not have a clear focus about the source of information used and the expert assessment. Moreover, not all types of treatments are always described, there is a little attention to the risks and benefits of each treatment and there is a not clear information about the long-term lifestyle changes.

In general, the websites dedicated to obesity treatment and weight loss should have better information about treatment and about the chronic nature of obesity that leads a long-term care and the importance of comprehensive approach to lifestyle change [35].

This is the first contribution, in our knowledge, that evaluates the quality of obesity treatment comparing different language websites to suggest and promote specific quality health information to orient Internet users all over the world. As in the other studies mentioned before, in general, the health information also in this specific topic doesn’t show a high level of quality information and easy readability on Internet websites worldwide.

It should be borne in mind that most websites, especially those in the English language, were dedicated to specific topics (pharmacotherapy or bariatric surgery) and were aimed at users that already were looking for something so specific. Their prominence in the rank of the search engine depended on algorithms that emphasize the number of searches, links to the page, and the number of clicks on the website by users. Nevertheless, since users often only take a look at the first records in a search, it is important that websites devoted to a health topic offer the most comprehensive list of information about that topic, to avoid a biased view by part of the users.

### Implications for the clinics

Considering the increasing rate of people with obesity and the impact of the online search for own health-related problems and about the choices of treatments, it is necessary having access to cheap and effective interventions [38]. To achieve this goal, it is important to improve the quality of online information and treatment accessibility [12]. Low quality of web information contributes to reducing the promotion of correct health information for internet users. To address a complex health condition like obesity, it is important to have access to information about all the different treatment options and on the lifestyle changes in the long term that are important to manage the condition. Poor quality of Internet information represents a limit for equal access to information about different treatments. Clinicians and Internet reviewers should be requested to write healthcare information in a manner accessible to all, with a clear definition of the goals and the risks of all treatments, also promoting shared decision making. According to the International Guidelines on the topic, the Internet users have to found clearly an emphasis on the different treatments related to the different conditions of obesity [24]. In particular, it should be always present the indication for a comprehensive lifestyle intervention, that includes building a skill set of behavioral knowledge and strategies to achieve and maintain dietary and physical activities in a long term [35]. The guidelines emphasize the chronic nature of obesity and the need for long-term care and also focus the objective of weight loss not for esthetic problems but for improving the health condition [7]. Dietary recommendations emphasize reducing intake of food and fat by 500–1000 cal/day. In addition, physical activity, varying from a minimum of 150 min a week to 300 min/week, is widely recommended. Recommendations concerning pharmacotherapy in most guidelines restricted its use to patients with BMI exceeding 30 kg/m^2^. Bariatric surgery is recommended when BMI > 40 for patients in whom other treatment approaches failed [34]. For each treatment, users have also to find the benefits and the risk about the treatment, a support to the shared decision making, the risk to postpone the treatment. It is also important to be sure that are valid and high-quality level that users can access to the sources used and that in the websites were made an editor medical review (Table 2 in ESM websites DISCERN criteria for the quality evaluation, that can help to find the correct information).

### Implication for research

To monitoring and support the process of online sharing of health information, it is important to define a clear evaluation methodology for the quality, accessibility and technological aspects of Internet information. It is as well important to monitor the quality of websites across countries to avoid that only English speakers can have access to high-quality information. For example, the adherence to HONcode certification is in general associated with websites of the highest quality [13], but Italian websites rarely adhere to the HONcode. Thus, it could be useful to create guidelines in different languages to promote a high standard for writing online health information both for professional and users.

### Strengths and limitations

The methodology that was used in this study is under development and for some of the tools there still some degree of uncertainty about the process of attribution of the score. Moreover, the intra-raters reliability of the JAMA Benchmark criteria was low, leaving open the question on the reproducibility of the evaluation based on this tool. However, even if this methodology is under development, we selected standardized and multidimensional methods for a global evaluation of the quality information. Of course, we need more studies to better define an international methodology.

### What is already known on this subject?

Internet is used as an important source of health information and as a tool for the sharing of medical knowledge. It may make patients better informed. But at this point, the quality information in general are inconsistent in terms of quality and also for the methodology of evaluation.

### What this study adds?

This study adds a multidimensional methodology for the quality evaluation specific for Internet health websites, suggest and inform Internet users in order to looking for a high-quality health information and also for the health workers to better improve the information available online according to the guideline about the treatment for Obesity condition. In the Digital Era, a good level of Internet health information it can be part of the prevention process and helps public health services.

## Conclusions

The quality of Internet information does not reach a high level worldwide. In the Digital Era, monitoring and evaluating the standard of online health information is an important scope of investigation, to ensure the rights of equal and high quality of health information access. This study is a first step to creating a multidimensional and standardized quality assessment of health information in the Internet that offer a useful tip for searching health information online.

## Supplementary Information

Below is the link to the Supplementary Information.

Supplementary Information 1 (DOCX 366 kb)
