# Supplementary material for: Quality of information of websites dedicated to obesity: a systematic search to promote high level of information for Internet users and professionals
Source: Eat Weight Disord. 2021 Mar 4;27(1):1–9. doi: 10.1007/s40519-020-01089-x (PMC8860948; doi:10.1007/s40519-020-01089-x)
Supplement: Supplementary file 1 [file 40519_2020_1089_MOESM1_ESM.docx]

Websites	Ita/ Eng	Reliability (clearly, relevant information, based on sources of information, expert assessment)	Quality of information (describe how each treatment works, each benefits and risk, treatment choice, support for shared decision making)	Overall quality ratin
https://www.endocrinologiaoggi.it/2018/06/terapia-farmacologica-dellobesita/	Ita	35	29	4
https://www.my-personaltrainer.it/farmaci-malattie/farmaci-obesita.html	Ita	28	23	3
https://www.fondazioneveronesi.it/magazine/articoli/alimentazione/dallobesita-si-puo-guarire-si-ecco-come	Ita	33	17	3
https://www.diabete.com/disponibile-anche-italia-la-nuova-terapia-orale-obesita/	Ita	34	22	4
http://www.quotidianosanita.it/scienza-e-farmaci/articolo.php?articolo_id=65769&fb_comment_id=18690798065	Ita	19	11	2
http://www.centro-obesi.com/come_si_cura_obesita.php	Ita	21	14	2
https://www.pharmamedix.com/patologiavoce.php?pat=Obesità&vo=Farmaci+e+terapie	Ita	38	30	5
https://www.starbene.it/approfondimenti/alimentazione/obesita-3187/cura-dell-obesita-3194	Ita	21	14	2
https://www.huffingtonpost.it/dottor-post/eccesso-ponderale-farmaci-per-l-obesita-a-che-punto-siamo_a_23431451/	Ita	26	25	3
https://www.vitalitaebenessere.it/dimagrimento-sovrappeso-e-obesita/?gclid=CjwKCAjwgbLzBRBsEiwAXVIygM-3y	Ita	17	10	2
http://www.lauraferrero.it/dietologia/nutrizione-clinica/obesita-sovrappeso/terapia-farmacologica/	Ita	21	17	3
https://www.centrodiriabilitazionenutrizionale.it/terapie1/obesita-adulti	Ita	15	9	2
http://www.societaitalianadiendocrinologia.it/html/news/liraglutide-obesita.asp	Ita	34	18	3
https://www.consultorioantera.it/approfondimenti/disturbi-del-comportamento-alimentare/obesita.html	Ita	20	18	3
https://www.messinamedica.it/2019/11/terapia-farmacologica-dellobesita-opzioni-terapeutiche-attualmente-disp	Ita	33	24	4
https://www.obesita.org/obesita-il-trattamento/	Ita	23	13	2
http://www.lauraferrero.it/obesita-sovrappeso-classificazione-terapia-dieta/	Ita	28	25	4
https://lamenteemeravigliosa.it/trattamento-psicologico-dellobesita/	Ita	20	14	2
http://medmedicine.it/articoli/farmacologia/farmaci-per-il-trattamento-della-obesita	Ita	19	12	2
https://www.istitutobeck.com/trattamento-obesita	Ita	19	14	2
https://www.epicentro.iss.it/obesita/obesita	Ita	25	14	3
https://it.wikipedia.org/wiki/Obesità	Ita	33	17	3
https://www.lobesita.com	Ita	17	12	2
https://www.obesita.com	Ita	31	18	3
https://www.centroitalianoperlapsiche.it/obesita-quale-trattamento-nutrizionale/	Ita	20	12	2
https://www.obesita.org/obesita-infantile-terapia/	Ita	19	15	2
https://www.my-personaltrainer.it/Sintomi/Obesita	Ita	23	15	3
https://www.saperesalute.it/obesita	Ita	19	11	2
http://www.salute.gov.it/portale/salute/p1_5.jsp?area=Malattie_endocrine_e_metaboliche&id=175	Ita	26	8	2
https://www.msdmanuals.com/it-it/professionale/disturbi-nutrizionali/obesità-e-sindrome-metabolica/obesità	Ita	38	31	5
http://www.cisiob.it	Ita	16	16	2
https://www.starbene.it/medicina-a-z/obesitÀ/terapia	Ita	21	19	3
http://www.farmacista33.it/nuovo-farmaco-antiobesita-disponibile-in-farmacia-/pianeta-farmaco/news--34330.html	Ita	22	10	2
https://www.notizieora.it/terapia-anti-obesita-brucia-calorie-senza-aumentare-lappetito/	Ita	13	9	1
https://www.ambulatorioanoressiabulimiaobesita.it/trattamento-sovrappeso-obesita/	Ita	20	10	2
https://www.informazionisuifarmaci.it/rimonabant	Ita	37	24	4
https://www.ok-salute.it/alimentazione/obesita-le-10-regole-dimagrire/	Ita	21	11	2
https://www.my-personaltrainer.it/alimentazione/esempio-dieta-obesita.html	Ita	25	17	3
https://www.donnamoderna.com/news/societa/come-ho-sconfitto-obesita-obesity-day	Ita	16	8	2
https://www.migliaccionutrizione.it/articoli/diete/sovrappeso-e-obesita-le-emozioni-per-dimagrire/	Ita	18	11	2
https://www.fondazioneveronesi.it/magazine/articoli/alimentazione/obesita-e-diete-ecco-come-si-riprende-il-peso-perso	Ita	24	7	2
https://www.huffingtonpost.it/2015/02/12/dieta-movimento-dimagrire_n_6670286.html	Ita	17	12	2
http://www.kousmine.org/obesita/	Ita	17	12	2
https://www.dcomedieta.it/i-consigli-per-dimagrire-di-un-ex-obeso/	Ita	18	14	2
https://www.gvmnet.it/press-news/news-dalle-strutture/gravi-obesi,-metodo-di-cura-innovativo-per-perdere.aspx	Ita	20	14	2
https://www.humanitasalute.it/prima-pagina-ed-eventi/67319-obesita-piu-si-ingrassa-piu-difficile-dimagrire-parere-esperto/	Ita	15	8	2
https://www.galileonet.it/obesita-bloccare-due-enzimi-potrebbe-aiutare-a-dimagrire/	Ita	23	7	2
https://www.investireoggi.it/fisco/perdere-peso-da-diritto-agli-sconti-fiscali-anti-obesita-incentivi-per-il-dimagrimento/	Ita	19	9	2
https://gazzettadelsud.it/speciali/salute-e-benessere/2019/07/09/obesita-una-scossa-al-cervello-permette-di-dimagrire-da97a73a-2df5-48ba-9d11-3e7653c55143/	Ita	15	10	2

https://www.donnamoderna.com/news/societa/come-ho-sconfitto-obesita-obesity-day	Ita	16	8	2
https://www.migliaccionutrizione.it/articoli/diete/sovrappeso-e-obesita-le-emozioni-per-dimagrire/	Ita	18	11	2
https://www.fondazioneveronesi.it/magazine/articoli/alimentazione/obesita-e-diete-ecco-come-si-riprende-il-peso-perso	Ita	24	7	2
https://www.huffingtonpost.it/2015/02/12/dieta-movimento-dimagrire_n_6670286.html	Ita	17	12	2
http://www.kousmine.org/obesita/	Ita	17	12	2
https://www.dcomedieta.it/i-consigli-per-dimagrire-di-un-ex-obeso/	Ita	18	14	2
https://www.gvmnet.it/press-news/news-dalle-strutture/gravi-obesi,-metodo-di-cura-innovativo-per-perdere.aspx	Ita	20	14	2
https://www.humanitasalute.it/prima-pagina-ed-eventi/67319-obesita-piu-si-ingrassa-piu-difficile-dimagrire-parere-esperto/	Ita	15	8	2
https://www.galileonet.it/obesita-bloccare-due-enzimi-potrebbe-aiutare-a-dimagrire/	Ita	23	7	2
https://www.investireoggi.it/fisco/perdere-peso-da-diritto-agli-sconti-fiscali-anti-obesita-incentivi-per-il-dimagrimento/	Ita	19	9	2
https://gazzettadelsud.it/speciali/salute-e-benessere/2019/07/09/obesita-una-scossa-al-cervello-permette-di-dimagrire-da97a73a-2df5-48ba-9d11-3e7653c55143/	Ita	15	10	2
https://www.percorsomediga.it/obesita-in-gravidanza/	Ita	19	11	2
https://www.my-personaltrainer.it/dimagrire/obesita.html	Ita	20	8	2
https://www.obesita.org/approccio-funzionale/	Ita	31	17	3
https://www.eurosalus.com/obesita/dimagrire-movimento	Ita	20	9	2
https://www.ok-salute.it/salute/obesita-tutti-gli-interventi-per-dimagrire-i-requisiti-e-le-complicanze/	Ita	23	12	2
https://www.my-personaltrainer.it/dimagrire/obesita-infantile.html	Ita	26	8	2
http://it.yourwebdoc.com/obesity.php	Ita	25	23	3
https://www.laleggepertutti.it/242421_obesita-come-dimagrire-con-la-chirurgia	Ita	21	17	3
http://casco.policlinicoumberto1.it/obesita/	Ita	15	9	2
https://www.msn.com/it-it/salute/alimentazione/obesità-e-sovrappeso-sono-una-malattia/ar-AAK1BWl	Ita	22	16	3
https://www.my-personaltrainer.it/dimagrire/obesita-androide-ginoide.html	Ita	27	9	2
https://www.pazienti.it/contenuti/malattie/obesita	Ita	24	20	3
https://www.wired.it/scienza/medicina/2018/08/28/obesita-farmaco-peso-cuore/	Ita	24	10	2
https://www.humanitas.it/malattie/obesita	Ita	16	10	2
https://www.niddk.nih.gov/health-information/weight-management/adult-overweight-obesity/treatment	Eng	35	25	4
https://www.mayoclinic.org/diseases-conditions/obesity/diagnosis-treatment/drc-20375749	Eng	36	25	4
https://www.medicalnewstoday.com/articles/323691#takeaway	Eng	28	25	4
https://www.ucsfhealth.org/conditions/obesity/treatment	Eng	25	28	4
https://www.nhs.uk/conditions/obesity/treatment/	Eng	29	25	4
https://www.hopkinsmedicine.org/health/treatment-tests-and-therapies/intensive-behavioral-therapy-for-obesity	Eng	18	15	2
https://www.therapytribe.com/therapy/what-is-obesity/	Eng	26	16	3
https://www.healthline.com/health/obesity/treatments-to-manage-obesity#takeaway	Eng	28	22	3
https://en.wikipedia.org/wiki/Management_of_obesity	Eng	37	25	4
https://www.health.harvard.edu/blog/obesity-is-complicated-and-so-is-treating-it-2018053013943	Eng	31	19	3
https://www.omicsonline.org/obesity-weight-loss-therapy.php	Eng	25	12	2
http://afc-ce.com/obesity-therapy-a-new-perspective/	Eng	25	24	3
https://en.wikipedia.org/wiki/Obesity	Eng	31	13	3
https://obesitycanada.ca/managing-obesity/	Eng	14	7	1
https://www.cms.gov/medicare-coverage-database/details/nca-decision-memo.aspx?&NcaName=Intensive%20Be	Eng	30	20	3
https://www.cnbc.com/2018/12/12/new-obesity-drugs-are-coming-but-doctors-and-insurers-are-hurdles.html	Eng	24	13	3
https://www.aota.org/about-occupational-therapy/patients-clients/childrenandyouth/obesity.aspx	Eng	16	12	2
https://www.nih.gov/news-events/nih-research-matters/benefits-moderate-weight-loss-people-obesity	Eng	32	16	3
https://www.webmd.com/diet/obesity/ss/slideshow-obesity-weight-loss-tips	Eng	22	14	2
https://www.heart.org/en/healthy-living/healthy-eating/losing-weight/extreme-obesity-and-what-you-can-do	Eng	25	18	3
https://www.cdc.gov/healthyweight/losing_weight/index.html	Eng	24	14	3
https://www.medicinenet.com/obesity_weight_loss/article.htm	Eng	36	32	5
https://www.theguardian.com/society/2019/jan/05/truth-obesity-five-fat-myths-debunked	Eng	26	13	3
https://www.onhealth.com/content/1/morbidly_obese_tips_for_losing_100_pounds_or_more	Eng	26	17	3
https://healthengine.com.au/info/lifestyle-changes-for-obesity-and-weight-loss	Eng	28	17	3
